# Supplementary material for: Association between oral microbiome and seven types of cancers in East Asian population: a two-sample Mendelian randomization analysis
Source: Front Mol Biosci. 2023 Nov 21;10:1327893. doi: 10.3389/fmolb.2023.1327893 (PMC10702768; doi:10.3389/fmolb.2023.1327893)
Supplement: Supplementary file 1 [file DataSheet1.ZIP › Supplementary Materials/MR plots for tongue/tongue═╝/Prostate cancer/pheno.2037_to_prostate cancer_forest.pdf]

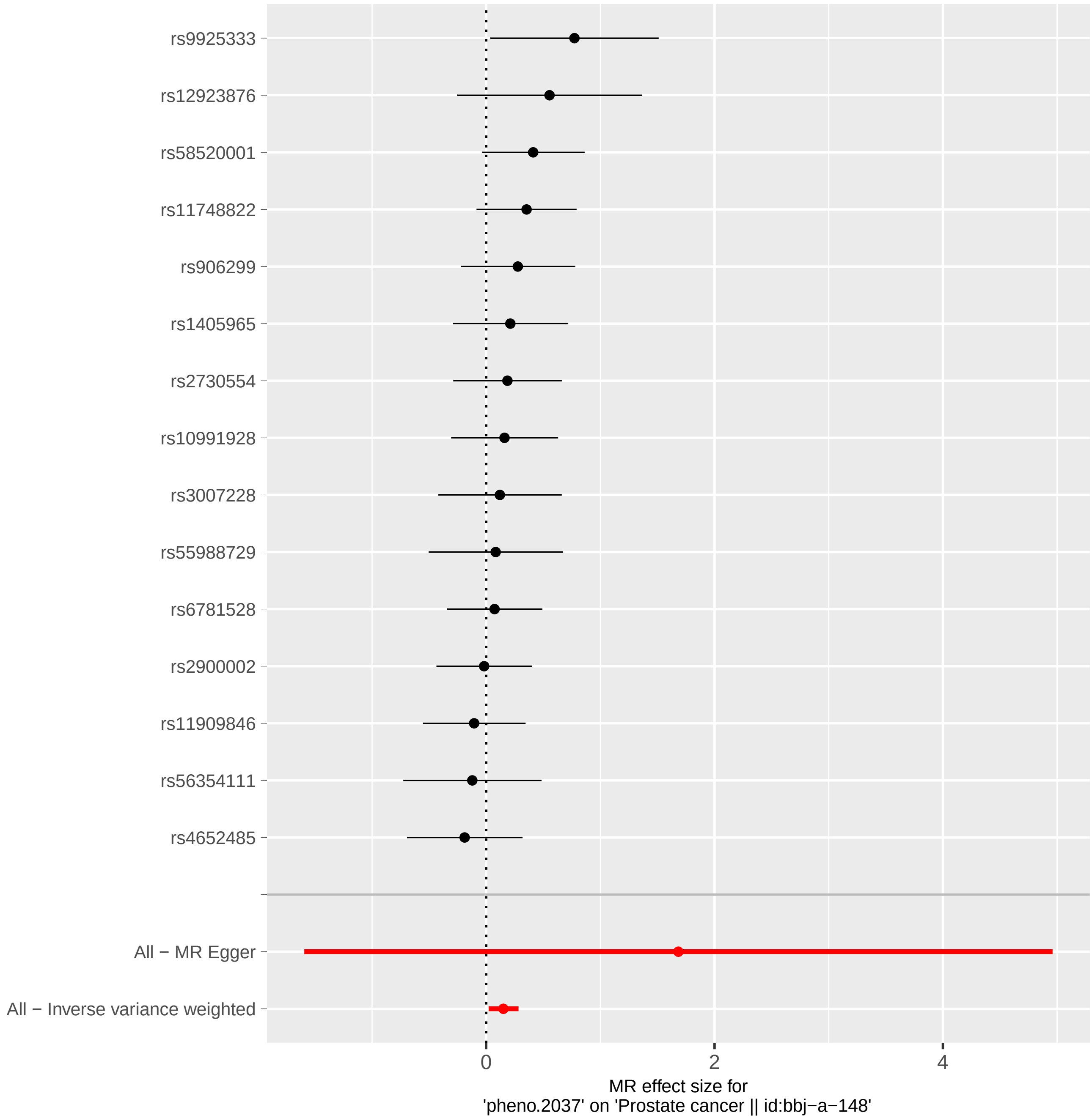

rs9925333
rs12923876
rs58520001
rs11748822
rs906299
rs1405965
rs2730554
rs10991928
rs3007228
rs55988729
rs6781528
rs2900002
rs11909846
rs56354111
rs4652485
All − MR Egger
All − Inverse variance weighted
0
2
4
MR effect size for
'pheno.2037' on 'Prostate cancer || id:bbj−a−148'
